# Climate Change and Autochthonous Vector-Borne Disease Transmission in Europe: Dengue as a Sentinel Signal for Surveillance and Preparedness

**DOI:** 10.3390/tropicalmed11070182

**Published:** 2026-06-29

**Authors:** Maciej Grzybek, Anna Bogacka

**Affiliations:** 1 Department of Tropical Parasitology, Institute of Maritime and Tropical Medicine, Medical University of Gdańsk, Powstania Styczniowego 9B, 81-519 Gdynia, Poland; 2 Department of Tropical and Parasitic Diseases, Faculty of Health Science with Institute of Maritime and Tropical Medicine, Medical University of Gdańsk, Powstania Styczniowego 9B, 81-519 Gdynia, Poland; anna.bogacka@gumed.edu.pl

**Keywords:** *Aedes albopictus*, arboviruses, autochthonous transmission, climate change, dengue, Europe, One Health, surveillance, vector-borne diseases

## Abstract

Climate change is reshaping the epidemiology of vector-borne diseases in Europe by altering the ecological conditions that determine vector survival, seasonal activity and pathogen transmission. Rising temperatures, milder winters, prolonged warm seasons and changing precipitation patterns are increasing the suitability of parts of Europe for competent mosquito, tick and sandfly vectors. These changes, combined with human mobility and land-use change, increase the probability that imported pathogens encounter permissive conditions for local transmission. This Opinion article examines autochthonous vector-borne disease transmission in Europe, using dengue as a sentinel example of a wider climate-sensitive transition. We discuss how imported viraemic cases, established competent vectors, vector–host contact and delayed clinical recognition can converge to enable local outbreaks. Beyond dengue, we consider West Nile virus, chikungunya, tick-borne encephalitis, leishmaniasis and Crimean–Congo haemorrhagic fever as examples of a broader and increasingly heterogeneous European risk landscape. We argue that the public-health impact of this transition is shaped not only by vector expansion, but also by gaps in surveillance integration, diagnostic readiness, workforce preparedness and One Health coordination. Strengthening climate-informed surveillance, rapid laboratory capacity, frontline clinical awareness and cross-sectoral response systems will be essential to prevent repeated introductions from becoming sustained public-health challenges.

## 1. Introduction

Climate change is reshaping the epidemiology of infectious diseases in Europe by modifying the ecological conditions that determine vector survival, seasonal activity, pathogen replication and human exposure. Rising mean and seasonal temperatures, milder winters, prolonged warm periods, altered precipitation and humidity, and changes in land use and urbanisation are increasingly favourable to several mosquito, tick and sandfly vectors [1,2]. These changes are occurring alongside international travel and population mobility, increasing the probability that imported pathogens encounter competent vectors during periods suitable for local transmission [2,3].

The European risk landscape is heterogeneous. Southern and Mediterranean regions currently experience the highest suitability for several Aedes-borne infections and leishmaniasis, whereas parts of central and northern Europe are increasingly affected by expanding mosquito and tick activity, including *Aedes albopictus*, *Ixodes ricinus* and, in some areas, introduced or sporadically detected *Hyalomma* spp. ticks [3,4,5,6]. This does not mean that Europe is becoming uniformly tropical. Rather, local climatic suitability, vector density, human exposure, diagnostic awareness and public-health response capacity interact to create geographically variable windows of transmission risk.

This Opinion article argues that climate-driven ecological change is transforming autochthonous vector-borne disease risk in Europe from a rare or exceptional phenomenon into a recurring preparedness challenge. Dengue is used as a sentinel example because it demonstrates how imported viraemic cases, established competent Aedes vectors and delayed clinical recognition can converge to produce local transmission. However, dengue is only one part of a broader climate-sensitive landscape that also includes chikungunya, West Nile virus, tick-borne encephalitis, leishmaniasis and Crimean–Congo haemorrhagic fever [7,8].

## 2. Climate-Sensitive Mechanisms of Vector Expansion and Local Transmission

Vector expansion should be interpreted not simply as a cartographic change in species distribution, but as a signal of altered ecological suitability. Temperature influences vector development, adult survival, biting activity and the extrinsic incubation period, defined as the interval between a vector acquiring a pathogen and becoming infectious. Warmer conditions may shorten this interval for some arboviruses, increasing the likelihood that infected vectors survive long enough to transmit the pathogen. At the same time, excessive heat, drought, reduced vector survival or unsuitable breeding habitats may limit transmission, emphasising that risk is nonlinear and context-dependent [2,7].

For Aedes-borne viruses, local transmission in mainland Europe requires the convergence of at least four conditions: introduction of the pathogen, usually by an imported viraemic traveller; presence of competent vectors such as *A. albopictus* or *A. aegypti*; climatic and environmental conditions that support vector activity and viral replication; and sufficient vector–human contact before the case is diagnosed and vector control is implemented [7]. Rainfall, humidity and the availability of water-holding containers influence larval habitats, while temperature governs the speed of viral replication within the mosquito [7].

*A. albopictus* is now established in many parts of southern and central Europe and continues to spread. ECDC and EFSA reported the species as established in hundreds of EU/EEA regions by June 2025, with new establishment or spread in several countries since the previous update [4]. *A. aegypti* remains much more geographically restricted in Europe, but its presence in selected areas and the possibility of reintroduction are important because it is a highly efficient vector of dengue, chikungunya, Zika and yellow fever viruses [5].

Tick and sandfly systems are affected through partly different mechanisms. For *I. ricinus*, warmer temperatures, milder winters, host abundance, vegetation and human outdoor activity influence seasonal questing and exposure risk. For *Hyalomma marginatum*, warmer summers and ecological suitability may increase the probability that immature ticks introduced by migratory birds survive to adult stages in new areas. Sandfly distribution and leishmaniasis risk are similarly influenced by temperature, humidity, host reservoirs and land-use change [2].

The major pathways by which climate-sensitive vector-borne diseases translate into preparedness needs are summarised in Table 1.

## 3. Dengue as a Sentinel Example of Autochthonous Arboviral Transmission in Europe

Dengue illustrates the transition from imported infections to locally acquired outbreaks in Europe. In mainland EU/EEA countries, dengue remains non-endemic and most cases are travel-associated. However, when viraemic travellers arrive in areas where competent Aedes vectors are established and active, local transmission can occur [7,9,10,11]. This makes dengue a useful sentinel for wider preparedness gaps because early clinical recognition, laboratory confirmation and vector-control measures must occur quickly enough to prevent amplification.

ECDC data indicate that from 2010 to 2024, 579 autochthonous dengue cases were reported in mainland EU/EEA countries involving transmission by Aedes albopictus, with a clear increase in recent years: 71 cases in 2022, 130 in 2023 and 304 in 2024 [7]. These numbers do not imply uniform European risk, but they demonstrate that recurrent local transmission is now plausible during favourable seasonal windows. The pattern is consistent with published reports from Italy, France, Spain, Croatia and Madeira/Portugal [10,11,12,13,14,15,16,17].

Selected milestones in European autochthonous dengue transmission are summarised in Table 2.

The 2024 outbreak in Fano, Marche Region, central Italy, illustrates how local transmission may be amplified by ordinary clinical uncertainty rather than by pathogen novelty alone. Early cases presented with non-specific febrile illness and were not immediately recognised as dengue in a setting without a history of sustained transmission. By the time dengue was confirmed, reactive vector control and intensified surveillance were required [14]. Similar lessons emerge from earlier Italian and French outbreaks, where recognition of local transmission depended on the coordination of clinical, laboratory, epidemiological and entomological information [11,12,13,16,17].

These events show that the distinction between imported and locally acquired disease is operationally important. In a warming Europe, travel history alone is no longer sufficient to rule in or rule out dengue and related arboviral infections. During periods of vector activity, clinicians and public-health authorities must consider both travel-associated and local exposure, especially in regions where *A. albopictus* is established.

## 4. Beyond Dengue: A Widening Spectrum of Vector-Borne Threats

Dengue is a sentinel signal, but not the only climate-sensitive vector-borne disease relevant to Europe. Chikungunya shares the Aedes-borne pathway and has already caused autochthonous outbreaks in Italy and France. ECDC historical data describe large outbreaks in Italy, including Emilia-Romagna in 2007 and central/southern Italy in 2017, as well as smaller French events [18]. These outbreaks demonstrate that preparedness for dengue, chikungunya and Zika cannot be separated from the distribution, density and seasonal activity of *Aedes* vectors.

West Nile virus represents a different ecological system. It is maintained in bird–mosquito cycles, mainly involving Culex mosquitoes, with humans and horses acting as incidental hosts. Climate affects mosquito abundance, viral replication and the timing of enzootic amplification, while bird migration and local ecology influence spatial spread. West Nile virus has become endemic or recurrently epidemic in parts of southern, central and eastern Europe, and modelling work has linked climate change to spatial expansion of risk [19].

Tick-borne encephalitis (TBE) is already established in many European countries, but its incidence and distribution are sensitive to climate, vegetation, host abundance and human outdoor exposure. ECDC reported 3516 confirmed TBE cases in the EU/EEA in 2022, with marked seasonality and most confirmed cases occurring between June and November [20]. Climate and land-use change may alter tick activity, altitude and latitude limits, and human exposure patterns, but interpretation requires caution because surveillance intensity, vaccination coverage and recreational behaviour also influence observed trends [2,21].

Leishmaniasis remains most strongly associated with southern Europe, but sandfly suitability may expand under warmer conditions. The risk is shaped not only by climate but also by reservoir hosts, especially dogs, wildlife ecology, peri-domestic environments and socioeconomic conditions. This makes leishmaniasis an important example of why One Health surveillance must include veterinary and environmental information, not only human case reporting [2,22].

Crimean–Congo haemorrhagic fever virus (CCHFV) represents a high-consequence scenario. The virus is transmitted primarily by *Hyalomma* ticks and maintained in tick-vertebrate cycles involving livestock and wildlife. Climate suitability for Hyalomma marginatum and related species is projected to increase in parts of Europe, and human cases have already been documented in some European settings. Although sustained transmission is not established across most of Europe, the severity of disease and biosafety requirements mean that preparedness must include diagnostic pathways, specimen transport procedures and clinical algorithms for suspected viral haemorrhagic fever [23,24,25].

## 5. Diagnostic and Workforce Capacity as Limiting Factors

The ability to detect and respond to emerging vector-borne infections depends critically on timely diagnosis and clinical recognition [26]. In many European settings, however, diagnostic and workforce capacity have not kept pace with the changing epidemiological landscape. Diseases historically regarded as rare or imported may not be routinely considered in primary care and emergency departments, where early recognition is essential for triggering public-health action.

Laboratory capacity is also uneven. Molecular and serological testing for dengue, chikungunya, West Nile virus, tick-borne encephalitis, leishmaniasis and CCHFV may be centralised in reference laboratories, creating delays between clinical suspicion and confirmation. For Aedes-borne viruses, the diagnostic window for direct viral detection is limited and serological cross-reactivity can complicate interpretation, particularly in populations with previous flavivirus exposure or vaccination [7,26]. Clinical guidance for post-travel fever and recent imported cutaneous leishmaniasis cases underline that correct travel and exposure history, timing of diagnostic tests, and awareness of atypical or treatment-refractory presentations remain essential for frontline readiness [27,28].

Workforce preparedness represents a parallel constraint. Many clinicians and public-health professionals in historically non-endemic areas have limited training in recognising and managing vector-borne diseases. This gap is particularly consequential during first local transmission events, when delayed suspicion can widen the window for mosquito exposure, secondary cases and reactive rather than preventive vector control.

Selected examples of European research and training capacity relevant to vector-borne disease preparedness are summarised in Table 3.

The objective is not to create a single European training model, but to ensure that frontline clinicians, diagnostic laboratories, entomologists, veterinarians and public-health teams share minimum operational competencies. These include recognition of compatible clinical syndromes, awareness of local vector activity, correct sample timing, rapid notification, and coordination with vector-control and environmental-health services.

## 6. Surveillance Integration and the One Health Gap

Effective preparedness for climate-sensitive vector-borne diseases depends on surveillance systems that can detect early signals across human, animal, vector and environmental domains. At present, surveillance in much of Europe remains fragmented: human case reporting, entomological monitoring, animal or reservoir surveillance and climate/environmental data are often collected by different institutions, using different objectives and reporting timelines [29,30,31,32,33,34]. This fragmentation limits the ability to translate early warning signals into preventive action.

Climate-informed surveillance requires more than counting human cases. It should combine vector distribution and abundance, pathogen detection in vectors or reservoirs, seasonal meteorological indicators, travel-related importation risk and syndromic or laboratory signals from clinical services. Digital surveillance, social-media monitoring, environmental data streams and modelling tools may contribute to earlier detection, but their value depends on data quality, interoperability and clear decision thresholds [29,30,31].

The One Health framework provides a practical basis for closing this gap. Operational One Health preparedness should link human clinical surveillance with veterinary reporting, wildlife or sentinel-animal data, entomological surveillance, environmental indicators and climate-informed risk assessment [32,33,34]. For West Nile virus, this means integrating human, equine, avian and mosquito data. For leishmaniasis, surveillance of dogs and sandflies is essential. For CCHFV, tick, livestock, wildlife and human health systems must be connected before a suspected human case appears.

A key challenge is governance. Data sharing across sectors may be constrained by institutional boundaries, different case definitions, incompatible databases and uncertain responsibilities for triggering action. Preparedness therefore requires agreed thresholds for intensified surveillance, vector control, public communication, blood-safety measures, laboratory referral and cross-border notification. Without such operational links, Europe will continue to detect many climate-sensitive events only after local transmission has already occurred.

## 7. Implications for Preparedness

The changing epidemiology of vector-borne diseases in Europe requires preparedness to be defined as adaptive system capacity rather than pathogen-specific emergency response. The aim is not to predict every outbreak, but to reduce the time between ecological signal, clinical suspicion, laboratory confirmation and public-health action [8,33,35].

First, surveillance should become seasonal, climate-informed and geographically targeted. Regions with established *A. albopictus*, recurrent West Nile virus activity, expanding tick-borne encephalitis risk or suitable *Hyalomma* spp. ecology require pre-season planning and rapid escalation pathways. Second, diagnostic systems should be prepared before the transmission season, including clear laboratory referral routes, guidance on specimen timing and algorithms for differentiating dengue, chikungunya, West Nile virus, TBE and other febrile syndromes. Third, workforce training should focus on practical recognition of locally acquired disease in patients without travel history when exposure occurs in a risk area during vector season.

Future preparedness should also incorporate projections without treating them as deterministic forecasts. Continued warming, longer warm seasons and changes in precipitation are likely to expand the periods and locations in which local transmission is possible, but the realised burden will depend on surveillance quality, vector control, vaccination where available, housing, land use, travel patterns and public behaviour. This uncertainty strengthens rather than weakens the case for flexible preparedness systems [2,29,34,35]. Recent traveller-vaccination guidance further emphasises that dengue and chikungunya vaccination decisions require destination-specific risk assessment, consideration of previous dengue exposure, vaccine authorisation, safety signals and access to diagnostic confirmation [36]. Surveillance-based analysis of mosquito-borne disease trends in Poland and Europe for 2018–2024 likewise supports the need to interpret imported cases, autochthonous transmission and regional surveillance capacity together when planning preparedness [37].

European systems are already moving in this direction through ECDC/EFSA vector maps, EU-level surveillance, reference laboratory networks, public-health guidance for locally acquired *Aedes*-borne diseases and One Health frameworks [3,4,5,7,32,34,35]. However, these capacities remain uneven. The central policy challenge is to ensure that climate-sensitive risk assessment becomes embedded in routine local and national public-health practice rather than activated only after outbreaks are detected.

## 8. Conclusions

Climate change is transforming the epidemiological landscape of Europe by altering the ecological baseline for vector survival, pathogen replication and seasonal transmission opportunity. Dengue provides a sentinel example of this transition, but the broader risk landscape also includes chikungunya, West Nile virus, tick-borne encephalitis, leishmaniasis and Crimean-Congo haemorrhagic fever.

The public-health consequences of this transition will be shaped not only by vector expansion, but also by diagnostic readiness, clinical awareness, surveillance integration and cross-sector response capacity. Europe does not need to become uniformly endemic for tropical or vector-borne diseases to face repeated local transmission events. Preparedness must therefore move beyond historical assumptions of endemicity [35,38] and toward flexible, climate-informed and One Health-oriented systems capable of detecting and interrupting transmission early.

## Figures and Tables

**Table 1:** Climate-sensitive vector-borne disease threats in Europe: vectors, mechanisms and preparedness implications. The table provides selected examples rather than a comprehensive risk ranking.

Disease/Pathogen	Main Vector/Reservoir System	Climate-Sensitive Mechanism	European Evidence/Significance	Preparedness Implication
Dengue virus	*Aedes albopictus*; *Aedes aegypti*	Imported viraemic travellers; competent Aedes vectors; temperature-dependent viral replication; water-holding urban breeding sites	Repeated autochthonous events in France, Italy, Spain, Croatia and Madeira/Portugal; marked increase in reported mainland EU/EEA cases after 2022	Rapid recognition of febrile illness during mosquito season; laboratory confirmation; targeted vector control; travel history and local exposure assessment
Chikungunya virus	*Aedes albopictus*; *Aedes aegypti*	Same Aedes-borne pathway as dengue; vector adaptation and warm-season transmission windows	Local transmission documented in Italy and France, including large outbreaks in Italy and smaller French clusters	Preparedness should be integrated with dengue and Zika surveillance because the vector and response pathways overlap
West Nile virus	*Culex* spp.; birds as amplifying hosts; humans and horses as incidental hosts	Warm summers, mosquito abundance, bird migration and ecological conditions supporting enzootic amplification	Endemic or recurrent seasonal transmission in parts of southern, central and eastern Europe, with interannual variability	Integrated human, veterinary, equine, avian and mosquito surveillance; seasonal risk communication and blood-safety measures
Tick-borne encephalitis virus	*Ixodes ricinus* and related ticks; wildlife hosts; humans exposed through tick bites or unpasteurised dairy	Warmer seasons, milder winters, vegetation, host density and recreational exposure affect tick activity and human contact	Established and expanding risk in parts of central, northern and eastern Europe; strong seasonality in reported EU/EEA cases	Vaccination policies in risk areas; clinician awareness; harmonised surveillance and public education on tick-bite prevention
*Leishmania* spp.	*Phlebotomus* spp.; dogs and wildlife reservoirs	Temperature and humidity influence sandfly survival, seasonality and range; reservoir ecology shapes transmission	Endemic in southern Europe with concern for northward expansion of vector suitability	Veterinary–human surveillance linkage; reservoir control; awareness of non-travel-associated cases in expanding risk areas
Crimean–Congo haemorrhagic fever virus	*Hyalomma marginatum* and other *Hyalomma* spp.; livestock and wildlife hosts	Warmer summers and ecological suitability may support survival of introduced *Hyalomma* ticks and enzootic cycles	Established or emerging concern in parts of south-eastern and southern Europe; high-consequence pathogen requiring preparedness	Tick surveillance; livestock and wildlife monitoring; biosafety-aware diagnostics; clinical algorithms for haemorrhagic fever evaluation

**Table 2:** Autochthonous dengue transmission in Europe, 2010–2025: Selected milestones and preparedness interpretation. The table summarises publicly available ECDC surveillance information and published outbreak reports, with emphasis on the transition from sporadic events to recurrent local transmission [7,10,11,12,13,14,15,16,17].

Period/Year	Countries or Areas	Interpretation for Preparedness
2010	Croatia and metropolitan France	Early autochthonous events demonstrated that local dengue transmission could occur in European settings where competent Aedes vectors were present.
2012–2013	Madeira, Portugal	Large island outbreak associated with *Aedes aegypti*; important example of European dengue outbreak potential outside mainland EU/EEA.
2013–2019	Mainly France and Spain	Sporadic local transmission events; public-health response remained oriented mainly toward imported cases and local vector control.
2020	Italy and France	First documented autochthonous dengue outbreak in Italy and additional French events; reinforced the role of *Aedes albopictus* in temperate Europe.
2022	France and Spain	Substantial increase in locally acquired cases; signal of expanding seasonal transmission opportunity.
2023	Italy, France and Spain	Multiple clusters including Lazio and Lombardy in Italy and events in France; increased need for genomic, entomological and clinical integration.
2024	Italy, France and Spain	Largest recent annual burden reported in mainland EU/EEA, including the Fano outbreak in Italy; underlined diagnostic and frontline preparedness gaps.
2025 and beyond	Risk areas with established Aedes vectors	Continued risk is expected where imported viraemic cases coincide with vector activity, suitable temperature and delayed recognition.

**Table 3:** Selected European research and training capacity relevant to vector-borne disease preparedness. The list is illustrative and not intended as a comprehensive inventory or ranking.

Institution/Initiative	Relevant Contribution
London School of Hygiene & Tropical Medicine, United Kingdom	MSc and diploma-level training in tropical medicine, public health and medical entomology; major research capacity.
Liverpool School of Tropical Medicine, United Kingdom	Long-standing tropical medicine institution with clinical, laboratory and vector-borne disease expertise.
Institute of Tropical Medicine Antwerp, Belgium	Postgraduate tropical medicine and infectious disease training; diagnostics and public-health expertise.
Swiss Tropical and Public Health Institute, Switzerland	Medical parasitology, infection biology, diagnostics, public-health and global-health research capacity.
University of Copenhagen/PREPARE4VBD, Denmark	European research initiative addressing vector-borne disease diagnostics, ecology, omics and environmental DNA-based surveillance.
Heidelberg University Hospital/Heidelberg Institute of Global Health, Germany	Vector-borne disease, geo-health, tropical medicine and international health training and research.
University of Naples Federico II, Italy	Mediterranean vector-borne disease research, including field diagnostics and tick/vector studies.
University of Bern, Switzerland	Molecular detection, field-based molecular surveillance and pathogen ecology relevant to vector-borne disease preparedness.
Institute of Maritime and Tropical Medicine, Medical University of Gdańsk, Poland	Tropical, maritime and travel medicine expertise; relevant capacity in central/eastern European preparedness.

## Data Availability

All data are presented within the manuscript.
